# Frequency and patterns of substance-induced psychosis in persons with concurrent mental health and substance use disorders during the COVID-19 pandemic: A Norwegian register-based cohort study

**DOI:** 10.1192/j.eurpsy.2024.1797

**Published:** 2024-12-16

**Authors:** Marja Leonhardt, Jørgen G. Bramness, Eline Borger Rognli, Lars Lien

**Affiliations:** 1 Norwegian National Advisory Unit on Concurrent Substance Abuse and Mental Health Disorders, Innlandet Hospital Trust, Brumunddal, Norway; 2 Faculty of Health Studies, VID Specialized University, Oslo, Norway; 3 Section for Clinical Addiction Research, Oslo University Hospital, Oslo, Norway; 4 Department of Alcohol, Tobacco and Drugs, Norwegian Institute of Public Health, Oslo, Norway; 5 Department of Clinical Medicine, UiT The Arctic University of Norway, Tromsø, Norway; 6 Faculty of Social and Health Sciences, Inland Norway University of Applied Sciences, Elverum, Norway

**Keywords:** comorbidity, COVID-19, mental health disorder, register study, substance use disorder, Substance-induced psychosis

## Abstract

**Background:**

Substance use may be associated with the onset of psychotic symptoms, necessitating treatment for individuals with comorbid mental health and substance use disorders (MHD/SUD). COVID-19 significantly impacted individuals with MHD/SUD, reducing access to appropriate care and treatment. Changes in drug availability and prices during the pandemic may have influenced drug consumption. This study aimed to determine the frequency of substance-induced psychosis (SIP) during COVID-19 among individuals with MHD/SUD and to explore substance fidelity by following patterns of SIP over time.

**Method:**

In this retrospective cohort study, we analyzed data from all individuals with MHD/SUD registered in 2019–2021 in the Norwegian Patient Register. We used graphical approaches, descriptives, and Poisson regression to study occurrence and risk of SIP episodes in the three-year observation period. Sankey diagrams were used to examine trajectories of psychotic episodes induced by various substances.

**Results:**

Despite a decrease in individuals diagnosed with SIP during COVID-19, SIP episodes increased overall. We observed a decline in cannabis-induced psychosis, but a rise in SIP episodes involving amphetamines and multiple substances. Among individuals with recurrent SIP episodes, the psychosis was more often induced by different substances during COVID-19 (2020: RR, 1.50 [95% CI, 1.34–1.67]; 2021: RR, 1.30 [95% CI, 1.16–1.46]) than in 2019.

**Conclusion:**

During COVID-19, fewer individuals were hospitalized with SIP, but those patients experienced more episodes. There were fewer cannabis-induced psychotic episodes, but more SIP hospitalizations caused by central stimulants and more SIP diagnoses caused by different substances, possibly reflecting changes in drug availability and pricing.

## Background

There is strong evidence of an association between substance use and the onset of psychotic symptoms [1, 2]. According to the Diagnostic and Statistical Manual of Mental Disorders (DSM-5), substance-induced psychosis (SIP) is a psychotic disorder that occurs during or soon after drug intake, causes hallucinations, delusions, and psychomotor disturbances, and is not better explained by other factors [3]. A similar description is found in the International Classification of Diseases (ICD) [4]. In Norway, around 500 patients are admitted every year to specialist health care due to an SIP [5]. As SIP occurs in close temporal association with substance use, the occurrence of SIP in the population may be sensitive to changes in use. For instance, cannabis-induced psychosis increased in Scandinavia up to 2016 [5], coinciding with a clear increase in the potency of cannabis products [6].

Individuals with co-occurring mental health and substance use disorders (MHD/SUD) have poorer treatment adherence, higher relapse rates, and more frequent hospital admissions [7] than those with only SUD or only MHD. They have poorer health and a higher risk of premature death compared to the general population [8, 9], often related to additional physical disorders receiving too little attention [10, 11]. Due to these complex challenges, persons with MHD/SUD are in need of comprehensive and readily available healthcare services and may be more vulnerable to changes in these [12].

In terms of treatment availability, the COVID-19 pandemic had a profound impact worldwide [13], particularly on individuals with MHD/SUD [14]. The pandemic exacerbated the complex challenges often faced by persons with MHD/SUD and created new barriers to recovery and well-being [15]. The pandemic control measures led to reduced and altered provision of mental health and addiction treatment services in high-income countries [16]. For example, in Norway, low-threshold facilities for persons with MHD/SUD were closed due to infection control measures during the first lockdown and many MHD/SUD patients admitted to long-term inpatient care were discharged to make space for COVID-19 patients [17]. Although most of the services were reinstated in Norway after the first lockdown in spring 2020, there was a 60% decline in the availability and delivery of detoxification services all over Europe [18]. Direct contact, particularly in group settings, was significantly limited or completely suspended for an extended period, as were individual appointments with therapists, doctors, or health and social workers. Alternative care such as telemedicine, with online group support and psychotherapy, was offered. To prevent virus spread due to traveling, substitution treatment programs offered more take-home doses [19] and doctors were allowed more flexibility in prescribing medication [20]. However, persons with opioid use disorders combined opioid-assisted treatment (OAT) with more illicit drug use [21].

The illicit drug market changed during COVID-19 with changes in price and availability of drugs affecting the way in which they were consumed [22]. Most research focuses on the change of drug use patterns according to frequency, rather than changes to a different substance [23, 24]. In the early stages of the pandemic, prices of synthetic drugs increased and availability of methamphetamine was reduced in the US [25]. In Europe, markets were disrupted during the initial lockdown, mostly due to closed borders and physical distancing which limited the street sale of drugs [26]. In Denmark, the first lockdown increased cannabis prices due to reduced availability [27], whereas the majority of cannabis, heroin, and cocaine users in a German study did not report increased prices or decreased availability of their preferred substance [28]. European wastewater analyses delivered heterogeneous results: in Amsterdam (The Netherlands) and Castellón (Spain), there was a decrease in the consumption of stimulants such as cocaine, amphetamine, and MDMA, whereas in Milan (Italy) and Utrecht (The Netherlands), higher consumption of these substances was observed in 2020 compared to previous years [29].

To our knowledge, there have been no studies exploring SIP during the COVID-19 pandemic. We hypothesize that COVID-19 with its restrictions and infection control measures reducing treatment availability affected the substance use patterns of persons with MHD/SUD, which might be reflected in the occurrence of SIP and type of SIP. This study aimed to determine the incidence of SIP diagnoses in persons with MHD/SUD, both any SIP and substance-specific SIPs, during the two years of COVID-19 and compare it to the preceding year. Additionally, we sought to explore whether individuals who had multiple episodes of SIP within a year had consistent or varying substance-specific SIP diagnoses, which could potentially indicate the degree of substance fidelity during the pandemic.

## Method

### Data sources

We conducted a retrospective cohort study and used the unique Norwegian 11-digit personal identifier to merge individual-level information from the Norwegian Patient Register (NPR) with census data administered by Statistics Norway. The NPR collects comprehensive data on specialist healthcare contacts and admissions in Norway, including referral dates, length of stay, diagnosis according to ICD-10, admission type, treatment codes, and outcomes (that is, discharge or death). The register data provided by Statistics Norway include a wealth of sociodemographic information, covering factors such as date of birth/death, gender, country of birth, educational level, and employment status.

### Study population

The sample comprised all individuals aged 18 years or older who were registered with an MHD/SUD between 2019 and 2021 in the Norwegian specialist healthcare services. The ICD-10, Chapter V was used to identify persons with MHD/SUD. Persons who were registered with F10-F19 as a main or secondary diagnosis with any other concurrent main or secondary F diagnosis (excluding organic, symptomatic, mental disorders (F00-F09) were defined as those with MHD/SUD. We identified 36,940 individuals with MHD/SUD within the period of interest.

### SIP episodes

We investigated episodes of psychosis recorded in the NPR as a main diagnosis and induced by the following substances: alcohol (F10.5), opioids (F11.5), cannabis (F12.5), sedatives (F13.5), cocaine (F14.5), amphetamines (F15.5), hallucinogens (F16.5), volatile solvents (F18.5), and multiple substances (F19.5). Consecutive recordings of SIP were considered as belonging to the same episode if there were fewer than five days between the recordings, while those separated by five or more days were considered separate SIP episodes.

### Analysis

We first used graphical approaches and descriptive statistics including the χ^2^ test to explore the characteristics of the study population and identify possible patterns in psychotic episodes induced by various substances during the observation period. To estimate the association between the outcome (SIP) and year, we calculated the relative proportions or relative risks and the corresponding 95% confidence intervals (95% CIs) using Poisson regression models [30]. We used the sandwich estimation method to generate robust standard errors [31]. For the purpose of Poisson regression, we computed a variable for each individual substance (x) which induced a psychosis (0 = no SIPx, 1 = SIPx) and used them as dependent variables in the Poisson regression models (Table 2). The category predictor variable was the year, using 2019 as a reference category. To study the trajectories of psychotic episodes induced by the different substances, we plotted data in a Sankey diagram. For this purpose, we identified individuals who had been diagnosed more than once with psychosis induced by different substances. The Sankey diagrams show only episodes of transition to another type of SIP, plotted by year. We also calculated the relative risk to estimate the association between being diagnosed more than once with the same or a different SIP and the respective years (Table 3). Therefore, we computed the outcome variable “multiple episodes” (0 = same type of SIP more than once, 1 = transition to another SIP) and defined all other cases (only one SIP diagnosis during the entire observation period) as missing. Statistical significance was set at p < 0.05. All analyses were conducted using Stata SE/18.0 and the Sankey version 1.71 for Stata.

### Ethics

The study was approved by the South-Eastern Norway Regional Committee for Medical and Health Research Ethics (reference number 158909) and the data protection office of Inland Hospital Trust (reference number 135540).

## Results

### Frequency of SIP

**Figure 1.**
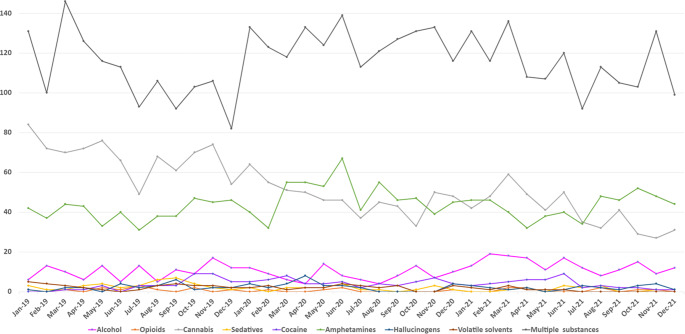
Monthly frequency of psychotic episodes induced by different substances in persons with MHD/SUD in 2019–2021.

From 2019 to 2021, we identified 1705 persons who experienced 8427 episodes of any SIP (Table 1). All SIP episodes were registered as a primary diagnosis. The most prevalent type of SIP every year was SIP induced by multiple substances (F19.5), followed by cannabis (F12.5) and amphetamine (F15.5). Figure 1 shows the development of the frequency of SIP episodes induced by different substances during the study period.

**Table 1.** Characteristics of the study population (N = 1705) and incidences of psychotic episodes induced by different substances by year

	2019	2020	2021	
	N (%)	N (%)	N (%)	Total N
Sex				
Male	456 (73.4)	399 (73.2)	387 (71.8)	1242
Female	165 (26.6)	146 (26.8)	152 (28.2)	463
Age (mean/SD)	33.9 (10.5)	34.0 (10.0)	34.8 (11.0)	34.2 (10.1)
SIP episodes				8427
Alcohol (F10.5)	118 (4.1)	103 (3.6)	163 (6.2)	
Opioids (F11.5)	12 (0.4)	5 (0.2)	8 (0.3)	
Cannabis (F12.5)	817 (28.2)	572 (19.8)	486 (18.5)	
Sedatives (F13.5)	38 (1.3)	19 (0.7)	13 (0.5)	
Cocaine (F14.5)	38 (1.3)	57 (2.0)	44 (1.7)	
Amphetamines (F15.5)	491 (16.9)	576 (19.9)	514 (19.5)	
Hallucinogens (F16.5)	24 (0.8)	31 (1.1)	23 (0.9)	
Volatiles (F18.5)	31 (1.1)	25 (0.9)	9 (0.3)	
Multiple substances (F19.5)	1332 (45.9)	1505 (52.0)	1373 (52.2)	
Trajectories of SIP				
Single episode	245 (8.5)	216 (7.5)	235 (8.9)	696^a^
> 1 episode with the same SIP	2221 (76.6)	2016(69.7)	1884 (71.6)	6121^b^
> 1 episode changing to another SIP (changers) from:	435 (15.0)	661 (22.9)	514 (19.5)	1610^c^
Alcohol (F10.5)	7 (1.6)	9 (1.3)	24 (4.7)	
Opioids (F11.5)	2 (0.4)	0 (0.0)	2 (0.4)	
Cannabis (F12.5)	149 (34.3)	89 (13.5)	64 (12.5)	
Sedatives (F13.5)	5 (1.2)	3 (0.5)	1 (0.2)	
Cocaine (F14.5)	17 (3.9)	42 (6.4)	14 (2.7)	
Amphetamines (F15.5)	69 (15.9)	171 (25.9)	102 (19.8)	
Hallucinogens (F16.5)	11 (2.5)	16 (2.4)	5 (1.0)	
Volatiles (F18.5)	0 (0.0)	0 (0.0)	0 (0.0)	
Multiple substances (F19.5)	175 (40.2)	331 (50.0)	302 (58.7)	

*Note:* All differences between the years were significant, with p < 0.001. All percentages are per year.

a In 696 individuals

b In 805 individuals

c In 204 individuals

SD = standard deviation; changer = person with more than one episode induced by different substances

### Association between psychotic episodes induced by different substances and year

Both the graph of the monthly frequency of SIP episodes (Figure 1) and the Poisson regression (Table 2) show a downward trend in cannabis-induced psychosis at the beginning of the pandemic, but an upward trend of amphetamine-induced psychosis from February 2020. There was a higher risk of amphetamine-induced psychosis in 2020 (RR, 1.18 [95% CI, 1.06–1.31] and 2021 (RR, 1.15 [95% CI, 1.03–1.29]) than in the prepandemic year. Similarly, there was a higher risk of psychosis induced by multiple substances during the pandemic than in the year before (2020: RR, 1.13 [95% CI, 1.01–1.19]; 2021: RR, 1.14 [95% CI, 1.07–1.20]). There was a lower risk of cannabis-induced psychosis in 2020 (RR, 0.70 [95% CI, 0.64–0.77]) and 2021 (RR, 0.66 [95% CI, 0.59–0.72]) compared to the prepandemic year. As for SIP due to alcohol, there was a higher risk of a psychotic episode in 2021 than in 2019.

**Table 2.** Association between SIP episodes induced by different substances and years

	Alcohol (F10.5)	Opioids (F11.5)	Cannabis (F12.5)	Sedatives (F13.5)	Cocaine (F14.5)	Amphetamines (F15.5)	Hallucinogens (F16.5)	Volatiles (F18.5)	Multiple substances (F19.5)
**Years**	RR (95% CI)	RR (95% CI)	RR (95% CI)	RR (95% CI)	RR (95% CI)	RR (95% CI)	RR (95% CI)	RR (95% CI)	RR (95% CI)
2019	1	1	1	1	1	1	1	1	1
2020	0.88(0.68–1.13)	0.42(0.15–1.18)	0.70(0.64–0.77)*	0.50(0.29–0.87)*	1.50(1.00–2.26)	1.18(1.06–1.31)*	1.30(0.76–2.20)	0.81(0.49–1.37)	1.13(1.07–1.19)*
2021	1.52(1.21–1.92)*	0.73(0.30–1.80)	0.66(0.59–0.72)*	0.38(0.20–0.71)*	1.28(0.83–1.96)	1.15(1.03–1.29)*	1.06(0.60–1.87)	0.32(0.15–0.67)*	1.14(1.07–1.20)*

*Note:* Modified Poisson regression with robust variance estimation.

N total episodes = 8427, RR = risk ratio.

* p < 0.05

### Trajectories of SIP

Most of the patients had an SIP several times per year (1009 patients [59.2% of the total sample] experiencing 7731 episodes) during the three-year observation period (Table 1). Among these, the majority (805 patients [79.8%] experiencing 6121 episodes) was diagnosed with the same type of SIP several times a year, and 204 patients (20.2%) with 1610 episodes were registered with different types of SIP diagnosis (here referred to as “changers”). As shown in Table 1, among the “changers,” there was an increase in patients initially registered with an amphetamine-induced psychosis who changed to psychosis induced by other drugs (15.9% in 2019, 25.9% in 2020, and 19.8% in 2021). A similar trend was seen in those with an initially recorded psychosis induced by multiple substances (40.2% in 2019, 50.0% in 2020, and 58.7% in 2021). There was a decrease in patients with initially recorded cannabis-induced psychosis changing to psychosis induced by other substances (from 34.3% in 2019 to 13.5% in 2020 and 12.5% in 2021).

Furthermore, the Poisson model showed that among patients with multiple SIP episodes, there was a higher risk of being a “changer” during the pandemic (2020: RR, 1.50 [95% CI, 1.34–1.67]; 2021: RR, 1.30 [95% CI, 1.16–1.46]) than in 2019 (Table 3).

**Table 3.** Risk of being a “changer” (being diagnosed with different types of SIP compared to the same type) by year

Year	N episodes	RR (95% CI)
2019	2611	1
2020	2644	1.50 (1.34–1.67)*
2021	2368	1.30 (1.16–1.46)*

*Note:* Modified Poisson regression with robust variance estimation; N total episodes = 7623, RR = risk ratio.; * p < 0.05 With “more than one SIP induced by the same substance” as a reference.

**Figure 2.**
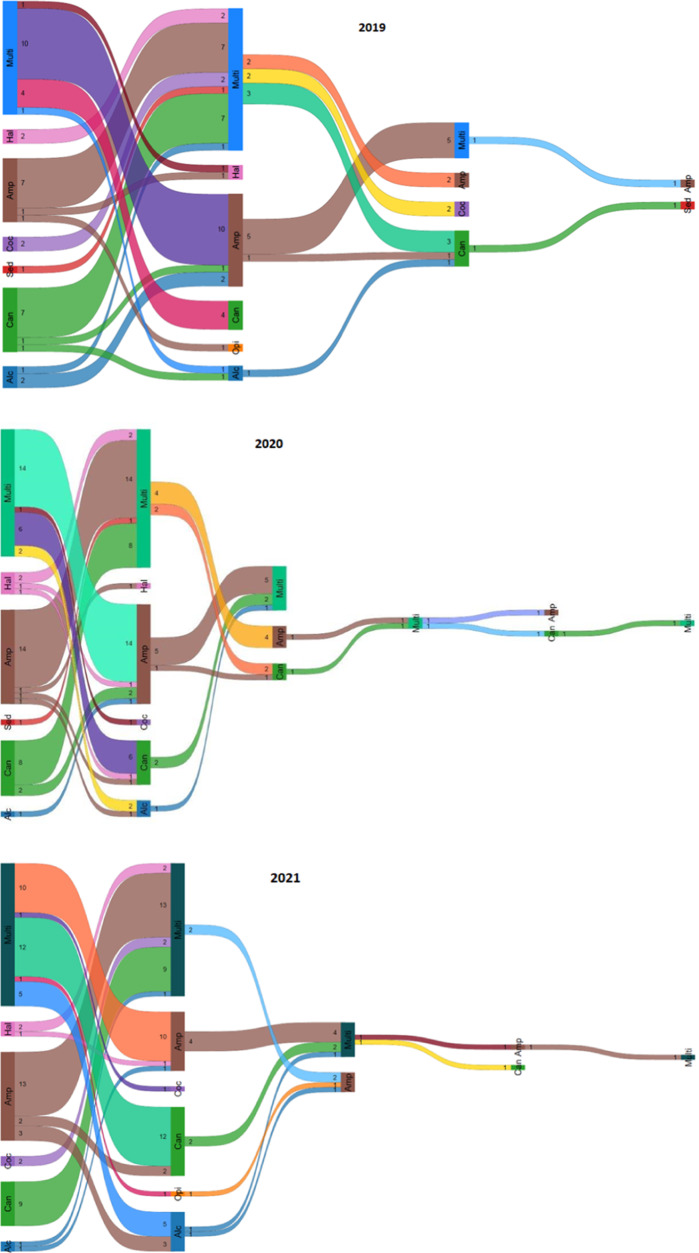
**(A–C):** Sankey diagrams showing the trajectories in persons diagnosed with more than one psychotic episode induced by different substances in 2019–2021. *Notes:* Alc, alcohol; Amp, amphetamines; Can, cannabis; Coc, cocaine; Hal, hallucinogens; multi, multi substances; Opi, opioids; Sed, sedatives; Number indicates the count of SIP episodes by 204 persons in total; SIP episodes induced by the same substance are not included.

The Sankey diagrams (Figure 2) show only the SIP episodes where patients switched from one substance to another, thus excluding episodes induced by the same substance. In 2019, there were 58 recorded episodes of such transitions, with some patients changing substances up to three times. In 2020, there were 76 episodes, with some patients switching up to five times, while 77 episodes were recorded in 2021, when some changed substances up to four times. Most transitions were from psychosis induced by amphetamine (2019:7; 2020:14; 2021:13) to psychosis induced by multiple substances and vice versa, from multiple substances to amphetamine (2019:10; 2020:14; 2021:10). There were only a few initial SIP transitions from cannabis to amphetamine (2019:1; 2020:2; 2021:0), and vice versa, with one episode in 2020 and two in 2021. Changers first diagnosed with psychosis induced by multiple substances showed six transitions in 2020 and twelve transitions in 2021 to psychosis induced by cannabis. Transitions from cannabis-induced psychosis to psychosis induced by multiple substances were recorded seven times in 2019, eight times in 2020, and nine times in 2021. Overall, there were few secondary transitions (14 in 2019, 15 in 2020, and 11 in 2021) or tertiary transitions (two in 2019, five in 2020, and three in 2021).

## Discussion

In this study, we explored the development of SIP episodes during the COVID-19 pandemic. Although the number of individuals with SIP decreased during the COVID-19 years, there was an increase in the number of episodes. There was a significant decline in cannabis-induced psychosis, but a rise in SIP episodes caused by amphetamine and multiple substances. Most people experienced more than one episode of SIP, but among those with several SIP episodes, most had several episodes triggered by the same drug. However, there was an increased likelihood of being diagnosed with different types of SIP during the two pandemic years 2020 and 2021 than in 2019. This involved more people changing from amphetamine-induced psychosis to psychosis induced by multiple substances and vice versa, but in fact fewer from cannabis-induced psychosis to psychosis induced by other drugs and vice versa, apart from the year 2021.

The pandemic with its restrictions exacerbated the health conditions of people already marginalized in society [32]. Thus, another explanation for the high number of SIP episodes during the pandemic years might be that those with SIP were already in a low socioeconomic position, with few alternative ways of adapting to restrictions, and a higher consumption of drugs, which may have led to more SIP episodes.

Interestingly, despite an overall decrease in the number of individuals experiencing SIP during the COVID-19 years, we observed an increase in the frequency of SIP episodes during the same period. Fewer individuals with more episodes could be viewed as a polarization of use, which has also been seen in alcohol use during the pandemic, where those with previous high consumption increased their use, while those with previous low or moderate consumption drank less alcohol [33].

Although many individuals with multiple SIP episodes had most of these episodes triggered by the same substance, there was a higher likelihood of individuals being diagnosed with different types of SIP during the pandemic than in the prepandemic year of 2019. People in Norway with concurrent MHD/SUD reported needing stronger drugs to cope with the pandemic, thus not showing fidelity to their usual drug or dosage [17]. Likewise, in a study from the UK, people who injected drugs reported changes in drug use patterns and transitions, especially to cocaine, benzodiazepine and pregabalin, during the pandemic [34]. These transitions to different drugs might reflect the occurrence of SIP and transition from one type of SIP to another, as our study revealed. Our data showed a decrease in SIP due to cannabis and an increase in SIP involving amphetamine and multiple substances in 2020, and this pattern was also observed in the changers, although the highest transition rate was to SIP involving multiple substances. Another reason for this development could be the changes in the drug market due to different types of restrictions. We also know that during the pandemic the illicit market was shifting toward more new psychoactive substances [35], the most prevalent of which were synthetic opioids, synthetic cannabinoids, and synthetic cathinones.

Our results may also reflect changes in general substance availability. Although the drug markets quickly rebounded after the onset of the pandemic, certain trafficking dynamics were strengthened during COVID-19, including larger shipment sizes, increased use of private aircraft, expanded use of waterway routes, and contactless drug transactions [36]. The reduced availability at the beginning of the COVID-19 pandemic and the associated higher price of certain drugs might also explain the significant decline in cannabis-induced psychosis and the concurrent rise in SIP episodes caused by amphetamine. Cannabis tends to have sedative effects, which may not have been desirable during periods of high stress or uncertainty. People may have sought the stimulating effects of amphetamines to combat the lethargy, low motivation, or depression associated with prolonged lockdowns and isolation [37].

Amphetamine-type stimulants are widely used as street drugs due to their affordability but have a high potential for addiction. Data from the Netherlands indicate that the number of methamphetamine laboratories increased from 9 in 2019 to 32 in 2020 [38]. The consumption of amphetamine in Europe has been rising since 2020 [39] and it has become more potent in recent years [40]. The narcotics report of the Norwegian police [41] reveals an extraordinarily high confiscation of amphetamines in 2020, which indicated a high circulation of this substance. The rise in production, potency and consumption could explain the rise of amphetamine-induced psychosis seen in our data.

The United Nations World Drug Report 2021 describes an increase in the use of cannabis globally during the pandemic [36]. European research also suggests an increase in cannabis consumption during the early months of COVID-19 [42]. However, Norwegian pandemic restrictions included closed borders at the beginning of the pandemic, which led to less smuggling of drugs and a cannabis drought, according to the narcotics report of the Norwegian police [41]. According to the Norwegian Organization for Humane Drug Policies, the price of cannabis in Norway rose 500 percent from 2019 to 2020 [43].

Our study shows that most SIP episodes were recorded as psychosis induced by multiple substances (F19.5), both before and during the COVID-19 period. This is consistent with previous research [5, 44-46], which found that individuals experiencing SIP were more likely to be involved in the abuse of multiple substances [47]. Also for substance users without a SIP diagnosis, polysubstance users are more likely to experience psychotic symptoms than those who predominantly use amphetamine or cannabis and alcohol [48]. Furthermore, many people diagnosed with F19.5 have probably used a mix of stimulants and a substance to end the binge or counter the effect of the stimulants, such as alcohol, benzodiazepines, or cannabis.

### Strengths and limitations

In this study, we included every person registered in the Norwegian specialist healthcare system with a concurrent MHD/SUD between 2019 and 2021, which enabled extensive coverage and robust statistical power, allowing for the analysis of subgroups, such as persons who experienced a SIP during those three years. Thus, we could analyze high-quality, routinely collected data, enabling us to study SIP outcomes over time. Furthermore, to our knowledge, this is the first study to explore trajectories of SIP. However, the study has some limitations which should be considered when interpreting the findings. First, it is difficult for clinicians to distinguish between SIP, primary psychotic illnesses, and psychotic illnesses with comorbid substance use, due to overlapping symptoms and complex patient histories [49]. Additionally, many individuals with primary psychotic disorders may use substances to self-medicate, which complicates the clinical picture further [50]. With the register data used in this study, we are not able to determine if the patients had a primary or secondary SUD before 2019. Hence, we were not able to use primary and secondary SUD separately as variables in the statistical analysis in a valid manner. There is also a potential selection bias due to the sample, which included only persons with a concurrent MHD/SUD. There is a likelihood of diagnostic overlap or misclassification in individuals with concurrent MHD/SUD. Symptoms of psychosis might be mistakenly attributed to substance use when they could be part of the underlying mental health disorder, or vice versa, leading to potential bias in identifying true cases of SIP. Moreover, the data collected during the COVID-19 pandemic may have been influenced by changes in healthcare seeking behavior and limited access to healthcare services, potentially impacting the data accuracy in relation to the number of SIP episodes. The study lacks a comparison group of individuals without MHD/SUD, which limits the ability to draw definitive conclusions about the specific impact of MHD/SUD on the SIPs recorded. Finally, the use of Sankey plots, which are based on aggregated data, may obscure individual variations and limit the granularity of the insights obtained from the analysis.

## Conclusion

Our study provides insights into the impact of the COVID-19 pandemic on the occurrence of SIP. While the number of individuals diagnosed with SIP decreased, the overall number of SIP episodes increased, particularly those caused by amphetamines and multiple substances, despite a decline in cannabis-induced psychosis. There were also more changes between different types of SIP, indicating that people had lower drug fidelity, and probably used what they could access. The findings imply that health professionals should be aware of the potential for an increased risk of SIP during crises such as the COVID-19 pandemic and maintain a high level of vigilance when assessing patients for substance use and related psychiatric symptoms.

## Data Availability

Access to the NPR can be requested through a standard data access procedure. Requests to access these datasets should be directed to https://helsedata.no/en/
